# Resveratrol‐Induced Suppression of C‐type Natriuretic Peptide Associates With Increased Vertebral Bone Density in Postmenopausal Women

**DOI:** 10.1002/jbm4.10732

**Published:** 2023-03-01

**Authors:** Timothy CR Prickett, Peter RC Howe, Eric A Espiner

**Affiliations:** ^1^ Department of Medicine University of Otago Christchurch New Zealand; ^2^ School of Biomedical Sciences and Pharmacy University of Newcastle Callaghan Australia; ^3^ Institute for Resilient Regions University of Southern Queensland Springfield Central Australia; ^4^ Adelaide Medical School University of Adelaide Adelaide Australia

**Keywords:** BONE TURNOVER MARKERS, C‐TYPE NATRIURETIC PEPTIDE, OSTEOPOROSIS, RESVERATROL

## Abstract

C‐type natriuretic peptide (CNP) is a paracrine growth factor essential in driving endochondral bone growth in mammals including humans. Despite evidence from animal experiments and tissues that CNP signaling stimulates osteoblast proliferation and osteoclast activity, whether CNP participates in bone remodeling in the mature skeleton is unknown. Using stored plasma samples from a previous randomized controlled clinical trial (RESHAW) of resveratrol supplementation in postmenopausal women exhibiting mild osteopenia, we have studied changes in plasma aminoterminal proCNP (NTproCNP) and concurrent change in bone turnover markers of formation (osteocalcin [OC] and alkaline phosphatase [ALP]) and resorption (C‐terminal telopeptide type 1 collagen [CTX]) with bone mineral density (BMD) over a 2‐year period of study in 125 subjects. In year one, subjects received placebo or resveratrol, switching to resveratrol or placebo, respectively, in year two. Across all time points, there were no significant associations of NTproCNP with CTX, ALP, or OC. During year one, plasma NTproCNP declined significantly in both groups. In the crossover comparison, analysis of change within individuals showed that, compared with placebo, NTproCNP declined after resveratrol (*p* = 0.011) and ALP increased (*p* = 0.008), whereas CTX and OC were unchanged. Inverse association of NTproCNP (*r* = −0.31; *p* = 0.025) and positive association of OC (*r* = 0.32, *p* = 0.022) with BMD at the lumbar spine were identified after resveratrol but not found after placebo. Decline in NTproCNP was independently associated with resveratrol treatment. This is the first evidence that CNP is modulated during a period of increasing BMD in postmenopausal women. Further study of NTproCNP and associations with drivers of bone formation or resorption can be expected to clarify CNP's role during other interventions directed to bone health in adults. © 2023 The Authors. *JBMR Plus* published by Wiley Periodicals LLC on behalf of American Society for Bone and Mineral Research.

## Graphical abstract

Estrogen lack or thyroid hormone excess increases vertebral tissue cytokines and increases CNP, which drives bone resorption (left). Resveratrol, or restoring normal thyroid hormone (right), reduces cytokine and CNP‐dependent bone resorption—and by augmenting bone formation increases bone density.

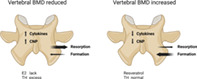

## Introduction

C‐type natriuretic peptide (CNP) is a paracrine growth factor expressed in numerous tissues including those of the vascular endothelium, neural, reproductive, and skeletal tissues.^(^ ^1^ ^)^ Among these, the role of CNP is most clearly established in growth plate skeletal cartilage, where the peptide has a crucial role in promoting endochondral growth of the immature skeleton.^(^ ^2^ ^)^ Although CNP gene transcripts are identified in osteoblasts,^(^ ^3^, ^4^ ^)^ periosteal stem cells,^(^ ^5^ ^)^ and osteoclasts,^(^ ^6^ ^)^ CNP's role, if any, in the adult skeleton remains unclear. In experimental animals, genetic models increasing CNP pathway activity stimulate markers of bone formation and resorption,^(^ ^7^, ^8^, ^9^ ^)^ but the impact of CNP on bone quantity and quality^(^ ^8^, ^9^, ^10^ ^)^ is controversial. In humans, rare genetic disorders resulting in gain^(^ ^11^, ^12^ ^)^ or loss^(^ ^13^ ^)^ of CNP activity show numerous skeletal anomalies and impaired bone quality in some reports both in overactive^(^ ^14^ ^)^ and reduced^(^ ^13^ ^)^ pathway activity. Although there is evidence of positive associations of plasma CNP products with bone turnover biomarkers in people,^(^ ^15^, ^16^ ^)^ no study has specifically examined CNP's participation during bone remodeling in older women at risk of metabolic bone disease.

A unique opportunity to evaluate CNP's role during changing bone mineral density (BMD) in people has recently been presented as part of the Resveratrol for Healthy Aging in Women (RESHAW) study. This randomized, double‐blinded, placebo‐controlled crossover interventional study^(^ ^17^ ^)^ examined the effect of the stilbenoid trans‐resveratrol, administered for 1 year as a supplement, on bone and metabolic health in postmenopausal women from the community. Compared with placebo, low‐dose resveratrol increased BMD in the lumbar spine and neck of femur and reduced bone resorption as reflected by decline in C‐terminal telopeptide type 1 collagen (CTX). Availability of appropriately stored samples of plasma from this study provided the means of determining putative roles of paracrine CNP during an active phase of contrived increase in bone formation. Two disparate hypotheses were postulated. Mindful that resveratrol upregulates CNP gene expression in human umbilical venous endothelial cells^(^ ^18^ ^)^ and has structural affinities with estrogen, which stimulates CNP in adult bone tissues,^(^ ^19^ ^)^ we first postulated that, compared with placebo, plasma aminoterminal proCNP (NTproCNP, a stable product of proCNP synthetized in tissues^(^ ^1^ ^)^) would be increased by resveratrol and would be positively associated with markers of bone formation. An alternative hypothesis, based on the negative impact of resveratrol on osteoclastic activity^(^ ^20^ ^)^ and the close association of declining plasma CNP products with CTX during correction of hyperthyroidism,^(^ ^16^ ^)^ predicts that plasma NTproCNP declines during resveratrol supplementation as markers of bone formation increase.

## Materials and Methods

As previously reported,^(^ ^17^ ^)^ this study examined the effects of resveratrol supplementation (75 mg twice daily) versus placebo on cognitive performance, cerebrovascular function, bone health, and cardiometabolic markers in postmenopausal women. The study, conducted at the Clinical Nutrition Research Centre of the University of Newcastle, Australia, was approved by the University Human Research Ethics Committee (H‐2016‐0091) and registered with the Australia and New Zealand Clinical Trials Registry (ACTRN12616000679482p).

Participants in whom appropriate plasma samples were available (125 aged 45 to 85 years, median 66 years at baseline) were enrolled between summer 2016 and winter 2017. All were community dwellers responding to recruiting radio and newspaper campaigns. To be included, participants were required to be amenorrheic for at least 12 months and not receiving estrogens for at least 3 months before enrollment. Median duration of menopause in women enrolled was 15 years (9 to 20 years). Exclusions were those receiving insulin or warfarin, or those with a history of breast or cervical cancer or major heart, kidney, liver, or neurological disorder. Participants were encouraged to maintain their usual habitual diet, including vitamin D or calcium supplement, and physical activity level throughout the study.

The study protocol has been described in detail^(^ ^17^ ^)^ but in brief constitutes a 24‐month randomized, double‐blind, placebo‐controlled, two‐period crossover intervention conducted to evaluate the effects of resveratrol supplementation (75 mg twice daily) on cognitive performance, cerebrovascular function, bone health, cardiometabolic markers, and well‐being in postmenopausal women.^(^ ^17^ ^)^ Group 1 received placebo in year one, followed by resveratrol in year 2, whereas the order was reversed in group 2. Metabolic and clinical measurements were made at baseline (screening) along with cognitive assessment and measures of cerebral blood flow—all repeated after year 1 and year 2. Venous blood (EDTA) samples were drawn after overnight fasting, centrifuged at 4000*g* for 10 minutes at 4°C, and plasma then separated, aliquoted, and stored at –80°C within 2 hours of collection. Bone turnover markers (BTMs)—alkaline phosphatase (ALP, Abbott ARCHITECT c16000, Abbott, Abbott Park, IL, USA), osteocalcin (OC, Roche Cobas e601, Roche Diagnostics, GmbH, Mannheim, Germany), and serum cross‐linked CTX (Roche Cobas e601)—were measured in a commercial laboratory (Pathology New South Wales). A separate (unused) aliquot available in all 125 completers was batched and sent frozen to the Christchurch Heart Institute Laboratory in New Zealand for measurement of NTproCNP as previously described.^(^ ^21^ ^)^ Intra‐ and interassay coefficients of variation for the NTproCNP assay at 31 pmol/L were 5.1% and 6.2%, respectively. All time points for a given individual were analyzed sequentially in the same assay.

Areal BMD was measured by dual‐energy X‐ray absorptiometry (DXA) in the lumbar spine (L_1_ to L_4_), left and right hip (total hip and neck of femur), as previously reported at baseline and at completion of each treatment arm. The integrated software (GE Healthcare [Chicago, IL, USA] Encore software v 16) was used to generate the BMD, *T*‐scores, and *Z*‐scores for the lumbar spine, hip, and whole body. Overall, the women were mildly osteopenic (femoral neck *T*‐scores at baseline between −1.0 and −2.5): specifically, 50 had normal bone density, 72 were osteopenic, and 6 had osteoporosis in the hips (*T*‐score <−2.5).

### Statistics

Data are presented as medians and interquartile range. For the cross‐over analysis, changes in values (delta) were calculated for individuals by subtracting the individual's 12‐month value from their value at 24 months. Within‐individual differences in NTproCNP circulating concentrations between resveratrol and placebo treatment phases were assessed by paired *t* tests. NTproCNP concentrations were log10‐transformed to satisfy parametric assumptions. Univariate associations between bone turnover markers or relevant clinical factors affecting bone health were analyzed using Spearman's correlation coefficients. Independence was assessed by multivariable regression analysis in a model comprising four separate variables—age, treatment group, 12‐month NTproCNP concentration, and delta ALP activity. All model assumptions were assessed graphically and log10‐transformed as appropriate to satisfy parametric assumptions. All tests were 2‐sided and statistical significance was assumed when *p* < 0.05.

## Results

Of the 125 participants completing the 2‐year trial, samples were available for analysis of NTproCNP in all at baseline and at 12 months. Samples at 24 months were unavailable in 8 participants: 7 in group 1 and one in group 2. Clinical details and other data (body mass index, blood pressure, BMD) have been published previously.^(^ ^17^ ^)^

NTproCNP and BTMs at baseline and after 12 and 24 months are listed for both groups in Table 1. A significant effect of time on plasma NTproCNP was observed for both groups (*F* = 144 and 114, respectively, *p* < 0.001 for both). As shown in Table 1, compared with baseline values, plasma NTproCNP was markedly reduced at 1 year for both those who received placebo and those who received resveratrol (*p* < 0.001 for both). CTX also fell during year one in those receiving placebo (*p* = 0.015) but not after resveratrol.

**Table 1:** Bone Turnover Markers (Median and Interquartile Range) at Baseline and 12 and 24 Months

	Baseline	12 months	24 months	
Group 1 (placebo year 1, resveratrol year 2)	*n* = 65	*n* = 65	*n* = 58	*p* Value
NTproCNP (pmol/L)	30.8 (24.9–35.7)	18.5 (16.9–21)	16.3 (14–18.7)	**<0.001**
CTX (ng/L)	431 (314–531)	370 (315–503)	413 (319–511)	**0.021**
ALP (U/L)	69 (58–82)	72 (61–84)	71 (61–80)	0.06
OC (μg/L)	19.4 (17.1–23.2)	19.1 (15.2–22.9)	18.6 (16.1–22.7)	0.22

Group 2 (resveratrol year 1, placebo year 2)	*n* = 60	*n* = 60	*n* = 59	
NTproCNP (pmol/L)	29.6 (24.2–37.8)	17.7 (15.8–20.6)	16.2 (14.5–19)	**<0.001**
CTX (ng/L)	458 (356–551)	442 (323–539)	446 (346–554	0.18
ALP (U/L)	73 (68–86)	77 (68–93)	74 (64–84)	0.06
OC (μg/L)	20.7 (16.4–23)	19.7 (14.3–23.6)	19.3 (14.3–23.8)	0.22

*Note*: Boldface text indicates *p* < 0.05.

Abbreviations: NTproCNP = aminoterminal proCNP; CTX = C‐terminal telopeptide type 1 collagen; ALP = alkaline phosphatase; OC = osteocalcin.

### Crossover analysis

Circulating concentrations of NTproCNP within individuals across the two‐period study were significantly lower (*p* = 0.039, *n* = 118) after resveratrol treatment (17.0 [14.8–19.6] pmol/L) compared with values after placebo (17.5 [15.5–20.2] pmol/L).

The impact on BTMs and NTproCNP of switching from placebo to resveratrol after 12 months in group 1 and the reciprocal switch in group 2 are shown in Table 2. Examining the change (delta) in each of the BTMs and NTproCNP after withdrawal or addition of the intervention (Table 2) shows that the decline in NTproCNP when receiving resveratrol was significantly greater than when receiving placebo (*p* = 0.011), whereas the change in ALP was significantly positive (*p* = 0.008). Change in CTX and OC was not significant. The relative strengths of variables that may contribute to these significant changes in NTproCNP and ALP were assessed separately using multivariable regression analysis (Table 3). For delta NTproCNP, the model incorporated age, NTproCNP at 12 months, resveratrol treatment (second arm crossover phase), and delta ALP (24 months minus 12 months prior). The results showed that delta ALP (*p* = 0.026), resveratrol treatment (*p* = 0.04), and NTproCNP at 12 months (*p* < 0.001) and age (*p* = 0.010) made independent contributions to the decline in NTproCNP in the crossover study. In a model predicting factors contributing to ALP increase during resveratrol treatment (second arm crossover phase), only resveratrol treatment (*p* = 0.049) and ALP at 12 months (*p* < 0.001) remained significant.

**Table 2:** Change in Bone Turnover Markers and NTproCNP (Delta) in Crossover Comparison

	Resveratrol (*n* = 57)	Placebo (*n* = 59)	*p* Value
NTproCNP (pmol/L)	−2.2 (−4.9 to −0.8)	−0.8 (−2.9 to 0.4)	**0.011**
CTX (ng/L)	−1.0 (−62.5 to 61.5)	−6.0 (−27.3 to 83)	0.24
ALP (U/L)	−2.0 (−6 to 5)	−5.5 (−11 to 2.3)	**0.008**
OC (μg/L)	−0.4 (−3.5 to 2.5)	−0.5 (−2.1 to 2.3)	0.44

*Note*: Boldface text indicates *p* < 0.05.

Abbreviations: NTproCNP = aminoterminal proCNP; CTX = C‐terminal telopeptide type 1 collagen; ALP = alkaline phosphatase; OC = osteocalcin.

**Table 3:** Multivariable Linear Regression Analysis—Crossover Comparison

	*B*	*SE(B)*	*β*	*t*	*Sig*.
Model 1: Dependent‐variable delta NTproCNP					
(constant)	1.2	2.6		0.47	0.64
Age	0.098	0.037	0.20	2.6	**0.010**
NTproCNP at 12 months	−0.476	0.058	−0.61	−8.2	**<0.001**
Delta ALP	0.062	0.0.28	0.17	2. 3	**0.026**
Resveratrol treatment	−1.19	0.58	−0.16	−2.1	**0.040**
Model 2: Dependent‐variable delta ALP					
(constant)	13.1	9.1		1.4	0.15
Age	−0.04	0.13	−0.03	−0.26	0.76
ALP at 12 months	−0.21	0.05	−0.38	−4.2	**<0.001**
Delta osteocalcin	0.25	0.18	0.12	1.4	0.18
Resveratrol treatment	3.8	1.9	0.18	2.0	**0.049**

*Note*: The coefficients of multiple determination (*R* ^2^) for models 1 and 2 were 0.44 and 0.21, respectively. Boldface text indicates *p* < 0.05.

Abbreviations: NTproCNP = aminoterminal proCNP; ALP = alkaline phosphatase.

### Associations with BMD

**Fig. 1:**
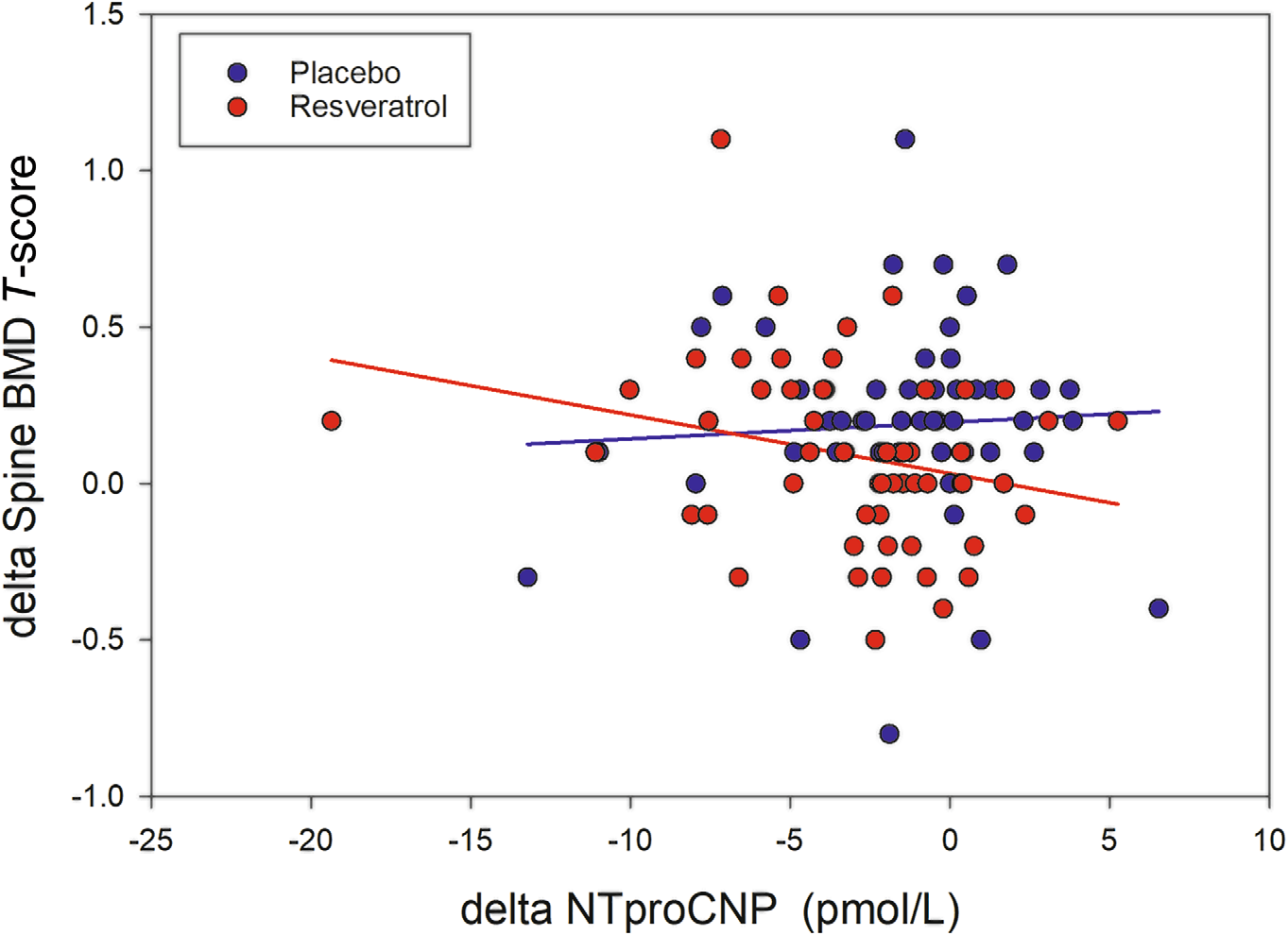
The relationship between change in bone mineral density (BMD) spine *T*‐score and change in plasma aminoterminal proCNP (NTproCNP) concentrations during the 12 months of the crossover arm after either placebo or resveratrol treatment.

At baseline, there were significant inverse associations of CTX and OC with lumbar spine *T*‐score (*R* = −0.28 and −0.21, respectively). Associations with NTproCNP and ALP were not significant. The strength and directions of these associations remained largely unchanged at 12 and at 24 months and were unaffected by either intervention. However, as shown in Table 4 in the crossover arm, within individuals there was a significant inverse association (*r* = −0.31, *p* = 0.025) between change in NTproCNP (fall) and BMD of the lumbar spine (increase) after resveratrol, not found after placebo (*r* = 0.09; Fig. 1). Along with the significant inverse association of NTproCNP with BMD, there was a significant positive association of OC (*r* = 0.32, *p* = 0.022) with BMD in the lumbar spine. No significant associations were found with BMD at the femoral neck, but at the hip, there was a significant inverse association of CTX with BMD (*r* = −0.42, *p* = 0.007) after resveratrol, not found after placebo (*r* = 0.09).

**Table 4:** Correlation of Change in BTMs and NTproCNP with Change in BMD *T*‐Score in the Crossover Comparison (*n* = 41–58)

	Lumbar spine		Femoral neck		Hip total	
	Placebo	Resveratrol	Placebo	Resveratrol	Placebo	Resveratrol
NTproCNP	0.09	**−0.31**	0.14	0.07	−0.07	0.04
CTX	**0.33**	0.24	0.02	−0.07	0.09	**−0.42**
OC	0.17	**0.32**	0.11	−0.22	−0.02	−0.09
ALP	−0.13	0.11	−0.03	−0.18	−0.14	0.07

*Note*: Significant correlation coefficients (*p* < 0.05) are highlighted with boldface text.

Abbreviations: BTM = bone turnover marker; NTproCNP = aminoterminal proCNP; BMD = bone mineral density; CTX = C‐terminal telopeptide type 1 collagen; OC = osteocalcin; ALP = alkaline phosphatase.

### Associations among BTMs and NTproCNP

Across all time points, there were no significant associations of NTproCNP with CTX, ALP, or OC. CTX was associated with ALP at baseline, 12 months, and 24 months (*r* = 0.41, 0.26, and 0.35, respectively) and with OC (*r* = 0.56, 0.62, and 0.60, respectively). Considering individual changes (delta) and combining results from placebo (*n* = 125), CTX was positively associated with OC (*r* = 0.43, *p* < 0.001) but not with ALP. The same relationships were found when results were combined after resveratrol. OC correlated with ALP after resveratrol (*r* = 0.28, *p* = 0.003) but not after placebo.

For each of the BTMs and for NTproCNP, associations of response to placebo with response to resveratrol were altered. Collectively this finding confirms the impact of resveratrol on biomarkers of both bone formation (OC and ALP) and resorption (CTX and NTproCNP).

## Discussion

Despite the well‐established anabolic and growth‐promoting role of CNP in the immature skeleton, whether CNP participates in maintaining bone health in the adult has not been studied. Here in this exploratory post hoc study, we show for the first time that plasma NTproCNP, a validated surrogate of CNP production in tissues,^(^ ^1^ ^)^ is significantly reduced during a period of demonstrable increase in BMD in postmenopausal women receiving resveratrol. Consistent with previous studies linking decline in plasma CNP products with contemporaneous increase in markers of bone formation during correction of a hypermetabolic state,^(^ ^16^ ^)^ the findings suggest a role of CNP in regulating osteoclastic activity associated with bone remodeling in humans.

### Change in NTproCNP and associations with resveratrol‐induced increase in BMD

Based on reports that resveratrol stimulates *NPPC* expression in human vascular endothelial cells,^(^ ^18^ ^)^ we first postulated that compared with placebo, plasma NTproCNP would be significantly increased by the stilbenoid. Supporting this hypothesis were reports that resveratrol has estrogen‐like activity in several tissues, including bone and data showing that estrogens stimulate CNP abundance in bone tissues and plasma.^(^ ^19^, ^22^ ^)^ Although findings during the first year of the study, in showing marked fall in plasma NTproCNP in both placebo and resveratrol interventions at 12 months complicate interpretation (as discussed below), results from analyses within each individual in the second year of the study clearly negate any stimulating action (first hypothesis) and in fact show that NTproCNP declines after 1 year of resveratrol treatment, supporting the second hypothesis. Notably, this change (decline) in NTproCNP is accompanied by a significant increase in ALP, whereas change in CTX was not significant (Table 2). Further, analysis of changes within individual subjects shows that the decline in plasma NTproCNP links with increase in BMD of the lumbar spine, not observed after placebo (Table 4). No previous study has examined change in CNP associated with change in BMD in individual subjects during contrived interventions affecting bone health. Interestingly, this inverse association was confined to the lumbar spine. The CNP association in the spine connects with studies in anestrus ewes where CNP abundance, compared with tibial tissues, was greater in vertebra, which was much more responsive to exogenous estrogen injections.^(^ ^19^ ^)^ The reduction in plasma NTproCNP (median 1.4 pmol/L), expressed as percentage of values at 12 months (18 pmol/L, 8%), is not trivial, bearing in mind the multiple tissues potentially contributing to levels in plasma.^(^ ^1^ ^)^ Notably, the increase in lumbar spine BMD (mean 4.7%) after resveratrol is similar to that found in meta‐analyses (4% to 5%) of the impact of 1 year of treatment with bisphosphonates,^(^ ^23^ ^)^ which was associated with 50% to 70% fall in markers of both bone resorption (CTX) and formation (PINP). In the current randomized controlled trial (RCT) using within‐subject changes over 2 years, there was an associated 7% to 8% decrease in NTproCNP, no change in CTX, and a 4.8% increase in ALP. Antiresportive drugs typically reduce all BTMs. Our study suggests that low‐dose resveratrol—inducing an increase in BMD comparable to that found with bisphosphonates—differentially impacts bone turnover by reducing resorption and increasing formation. Exactly similar directional responses in NTproCNP (fall), ALP (rise), and PINP (rise) occur during the course of correcting metabolism in subjects with hyperthyroidism.^(^ ^16^ ^)^ Collectively these directional changes in NTproCNP during bone repair in two quite different clinical settings confirm CNP's participation in bone remodeling and raise the possibility that CNP could be a unique marker of vertebral bone status. Intriguingly, T3 excess and E2 deficiency also share commonalities in regulating bone formation by stimulating NO cGKii pathway activity.^(^ ^24^ ^)^ Notwithstanding the reported positive impact of CNP on osteoblastogenesis in vitro^(^ ^4^, ^25^ ^)^ and in vivo,^(^ ^26^ ^)^ with respect to CNP actions in bone, our findings suggest that actions modulating resorption predominate, at least in settings of increased bone turnover in women associated with hyperthyroidism or estrogen deficiency. At the microcellular level during fracture repair, these actions of CNP may be crucial to callus resorption, which is intimately coupled to bone formation.^(^ ^8^ ^)^ Whether CNP is a driver of resorption or simply coupled to other factors participating in bone remodeling is unclear. Our study was not designed to address the underlying mechanisms whereby CNP impacts bone metabolism. Notably however, in both hyperthyroidism and estrogen deficiency, bone tissue abundance of a range of cytokines is increased.^(^ ^27^ ^)^ Cytokines potently increase *Nppc* expression in vascular and bone marrow tissues.^(^ ^28^, ^29^ ^)^ *Nppc* and its receptor NPRB are expressed in osteoclasts^(^ ^6^ ^)^ and in callus during remodeling of fracture repair.^(^ ^8^ ^)^ CNP stimulates bone resorption in mouse bone marrow cultures by activating existing osteoclasts rather than stimulating their formation.^(^ ^6^ ^)^ Further, in tibial tissue extracts excised from 12‐week‐old adult mice, CNP potently upregulates expression of several genes associated with osteoclast activity including sclerostin,^(^ ^9^ ^)^ a recognized inhibitor of bone formation. Plausibly, these findings suggest that the anti‐inflammatory actions of resveratrol^(^ ^30^ ^)^ reduce cytokine‐dependent CNP gene expression, reduce bone resorption, and, in complementing anabolic actions of the stilbenoid, enhance bone formation. Alternatively, the decline in CNP is simply a consequence of changing BMD and not itself driving resorption. Clearly, further study of CNP's role and regulation is required in this and other settings of changing bone health, including its efficacy as a biomarker of bone resorption in clinical studies during therapeutic interventions.

### High NTproCNP at baseline and progressive decline

Findings of (i) markedly raised concentrations of NTproCNP at enrollment and (ii) progressive fall in levels in the first year of the study, independent of intervention, were unexpected and need consideration in light of possible factors impacting circulating levels in this cohort before enrollment. Compared with concentrations in an age‐ and sex‐matched reference population drawn from healthy volunteers in the community,^(^ ^15^ ^)^ values at enrollment were almost 3 standard deviations higher, falling to closely similar levels well within the reference range in both groups 1 year later. Since a similar but much smaller decline across the second year occurs in both group 1 and group 2 participants, biological rather than technical factors are likely to be at play. Excepting the significant decline in CTX after placebo in year one, these changes were not identified in other BTMs. At baseline, no association of NTproCNP was found with a range of clinical variables^(^ ^1^ ^)^ potentially impacting circulating levels in community dwellers, excepting an inverse association with HDL cholesterol, which is a consistent finding observed in previous studies in middle‐aged subjects.^(^ ^31^ ^)^ It is well recognized that changes in participant's knowledge, habits, lifestyle, and intake of self‐prescribed health products on entering RCTs can affect a range of physiological variables.^(^ ^32^ ^)^ For example, in the current study, intake of dietary supplements, change in physical activity, and closer adherence to vitamin D/calcium intake have the potential to affect bone turnover. Since all participants were postmenopausal and overall exhibited mild osteopenia, increased bone turnover but variable rates of unit activation frequency^(^ ^33^, ^34^ ^)^ could be factors along with seasonal changes^(^ ^35^ ^)^ affecting levels at enrollment but are unlikely to contribute alone to the decline at 1 year. However, antecedent vertebral stress including subclinical fracture—a strong possibility in this cohort^(^ ^36^ ^)^—cannot be excluded particularly in light of the abundance of CNP in vertebrae, which links with increase in plasma NTproCNP during interventions in experimental animals.^(^ ^19^ ^)^ Future study employing serial NTproCNP sampling over 1 to 2 years in postmenopausal women, comparing values in those with and without osteopenia/porosis, can be expected to answer this question.

### Limitations and strengths

There are several caveats to consider in this post hoc study. The rationale for the original study (RESHAW) was directed to the impact of resveratrol on cerebral blood flow and cognition; hence, optimizing conditions for measuring NTproCNP in retained blood samples and assessing its potential impacts on bone health were secondary considerations. Thus, the strict conditions required for sampling and storage before assay of NTproCNP may not have been met. In the event, suitably collected (EDTA) and stored samples (up to 6 years) were available and sent to the source laboratory under carefully controlled conditions. Loss of immunoreactivity with storage at less than –20°C in our experience is <5% and would affect all samples similarly. The possibility that technical factors affecting the assay (hemolysis, batching, order of assay, etc.) could explain the raised levels at enrollment were all excluded, as was any effect of anticoagulants other than EDTA. Further, associations between plasma NTproCNP and BMD were modest and confined to the spine. These, together with the inconsistent changes in BTMs, underline the need for prospective studies specifically focusing on CNP products and approved BTMs during the course of recognized therapies that benefit bone metabolism in clinical practice.

None of the above limitations affects the main findings, viz that at low dose, (i) resveratrol significantly decreases plasma NTproCNP and (ii) the decline in NTproCNP is inversely correlated with increase in BMD of the lumbar spine in postmenopausal women. These findings have clinical significance for at least two reasons. First, no previous study has shown that CNP participates in bone remodeling in humans. Second, the findings raise the possibility that NTproCNP could serve as a key biomarker of bone remodeling, which, in view of the deficiencies in current techniques assessing bone resorption, needs to be addressed. More importantly, measuring NTproCNP in defined settings and during specific interventions in subjects with metabolic bone disease may add clarity to the molecular events underpinning changes in bone formation and resorption. In contrast to recognized BTMs, which reflect downstream activity of multiple proteins within a variety of skeletal tissues,^(^ ^37^ ^)^ the CNP signaling pathway has the potential to drive new bone formation^(^ ^25^, ^26^ ^)^ and modulate resorption.^(^ ^6^ ^)^ The relative importance of these effects during interventions impacting CNP activity could be assessed, for example, by measuring concurrent changes in sclerostin, RANKL, and osteoprotegerin.^(^ ^9^ ^)^ Change in the latter ratio—if associated with directional change in NTproCNP—would be expected to clarify CNP's role, especially if linked with changes in BMD and bone quality. The positive findings from this exploratory study can therefore be expected to lead to new advances in monitoring and treating subjects at risk of deterioration in bone quality.

## Author Contributions

**Timothy CR Prickett:** Conceptualization; formal analysis; writing – review and editing. **Peter RC Howe:** Conceptualization; data curation; writing – review and editing. **Eric A Espiner:** Conceptualization; writing – original draft; writing – review and editing.

## Disclosures

The authors declare no conflicts of interest.

### Peer Review

The peer review history for this article is available at https://publons.com/publon/10.1002/jbm4.10732.
